# Unraveling the Molecular Basis for G‐Quadruplex‐Binders to ALS/FTD‐Associated G4C2 Repeats of the *C9orf72* Gene

**DOI:** 10.1002/cbic.202400974

**Published:** 2025-01-20

**Authors:** Luisa D'Anna, Darren Wragg, Daniela Mauro, Simona Rubino, Alessio Terenzi, Giampaolo Barone, Sophie R. Thomas, Angela Casini, Riccardo Bonsignore, Angelo Spinello

**Affiliations:** ^1^ Department of Biological, Chemical, and Pharmaceutical Sciences Technologies, Università di Palermo Viale delle Scienze Edificio 17 90128 Palermo Italy; ^2^ Chair of Medicinal and Bioinorganic Chemistry School of Natural Sciences Department of Chemistry Technical University of Munich (TUM) Lichtenbergstr. 4 85748 Garching b. München Germany; ^3^ Department of Inorganic Chemistry University of Vienna Währinger Straße. 42 Vienna Austria

**Keywords:** G-quadruplex, C9orf72, molecular dynamics, docking, neurodegenerative disorders

## Abstract

The most recurrent familial cause of amyotrophic lateral sclerosis (ALS) and frontotemporal dementia (FTD) is the presence of an abnormal number of intronic GGGGCC (G_4_C_2_) repetitions in the *C9orf72* gene, which has been proposed to drive ALS/FTD pathogenesis. Recently, it has been shown that such G_4_C_2_ repetitions can fold into G‐quadruplex (G4) secondary structures. These G4s have been selectively stabilized by small‐molecule binders, furnishing proof‐of‐principle that targeting these non‐canonical nucleic acid sequences represents a novel and effective therapeutic strategy to tackle neurodegenerative disorders. However, precise information on the mechanism of action of these compounds is still lacking. Here, by performing *in silico* investigations, we unraveled the molecular basis for the selectivity of a series of known structurally related *C9orf72* G4‐binders. Moreover, we investigated the binding properties of a strong and selective metal‐based G4 stabilizer, the Au^I^ bis‐N‐heterocyclic carbene (NHC) complex – Au(TMX)_2_ – showing that it moderately stabilizes G_4_C_2_ G4 RNA by Förster resonance energy transfer (FRET) DNA melting assays. Using metadynamics (metaD) simulations, the Au(TMX)_2_ binding mode and the associated free‐energy landscape were also evaluated. This information paves the way for developing improved compounds to tackle ALS/FTD neurodegenerative disorders.

## Graphical abstract

Some neurodegenerative disorders can be driven by recurrent mutations in the *C9orf72* gene. These sequences can fold into G‐quadruplex secondary structures. Here, by performing *in silico* investigations coupled with FRET DNA melting assays, we unraveled the molecular basis for the selectivity of a series of organic and organometallic *C9orf72* G4‐binders.

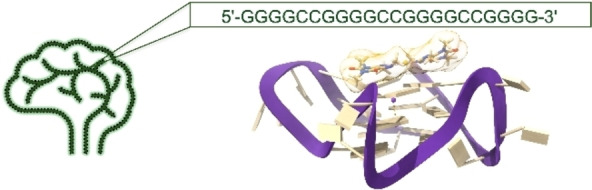

## Introduction

Repeated expansions of nucleotide sequences in neuronal genes represent the most recurrent molecular basis of neurodegenerative disorders. As an example, the expansion of GGGGCC (G_4_C_2_) repeats within the first intron of chromosome 9 open reading frame 72 (*C9orf72*) gene is the most frequent familial cause of two devastating neurological disorders: amyotrophic lateral sclerosis (ALS) and frontotemporal dementia (FTD).^[1]^ While healthy individuals carry less than 20 hexanucleotide repeat expansions (HRE), abnormal expansion, ranging from 500 to 4000 units of G_4_C_2_ repeats, is observed in ALS/FTD patients.^[2]^ However, the mechanism of how HRE drives ALS/FTD pathogenesis remains still elusive. Among the putative underlying mechanisms, HREs can be transcribed, and the resulting RNA sequences can form RNA foci that can sequester RNA‐binding proteins.^[3]^ Moreover, these sequences can be translated through a non‐canonical process called repeat‐associated non‐ATG translation, producing toxic dipeptide repeat (DPR) proteins.^[4]^

(G_4_C_2_)_n_ sequences can fold into distinct conformations, including DNA and RNA G‐quadruplexes (G4).[^5^, ^6^] These are stable four‐stranded structures composed of tetrads of guanines (G‐quartets), stabilized by cations, typically potassium ions in the central channel, and held together by Hoogsteen hydrogen bonds (H‐bonds).^[7]^ It has been shown that (G_4_C_2_)_3_G_4_ DNA sequences are able to fold into two major G4 structures showing an antiparallel topology and composed of four G‐quartets. These two structures were solved using solution‐state NMR and were named “neutral and annealing” (NAN) and “acidic and quenching” (AQP).[^8^, ^9^] Among these, NAN is thermodynamically favored, while AQP is kinetically favored under slightly acidic conditions. Regarding its RNA analogs, r(G_4_C_2_)_3_G_4_ was reported to fold into parallel G4 topologies.^[10]^ G4‐RNAs were usually reported to adopt preferentially a parallel fold as a consequence of the anti‐conformation assumed preferentially by riboguanosine. However, it was recently observed that RNA G4s are not more prone to fold into a parallel topology compared to what was observed for DNA G4s.^[11]^ Unfortunately, structural data on r(G_4_C_2_)_n_ sequences are still lacking to this date.

Putative G4‐forming sequences have been observed in many regions of the human genome, such as in chromosomal DNA and transcribed RNAs. Thus, the stabilization of G4s by small molecules was proposed as a potential therapeutical strategy to modulate gene expression and immune gene response.^[12]^ Among these binders, the cationic porphyrin TMPyP4 has been previously shown to bind and stabilize several distinct G4 structures.^[13]^ Moreover, it was recently shown that TMPyP4 can bind and alter the secondary structure of r(G_4_C_2_)_n_ sequences.^[14]^ Metal‐based compounds offer additional features as compared to organic molecules, such as distinct redox activity, reversible coordinating bonds, and a wider variety of different coordination geometries. Thus, they represent an alternative platform to bind nucleic acids and, in particular, G4 structures.[^15^, ^16^] Successful examples are represented by metalloporphyrins,^[17]^ salphen complexes,[^18^, ^19^, ^20^] and gold complexes.^[21]^

**Figure 1:**
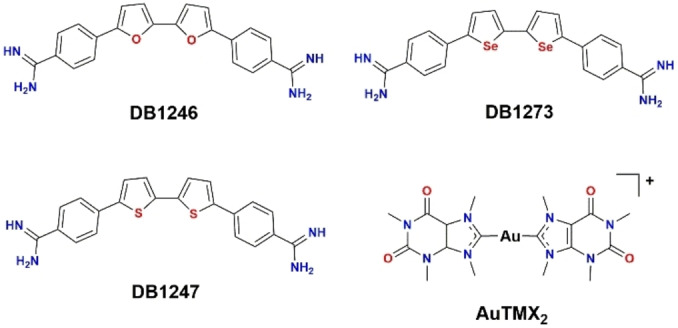
Molecular structures of DB1246, DB1247, DB1273 and Au(TMX)_2_.

In this context, Simone et al. recently identified three structurally related compounds (DB1246, DB1247, DB1273, Figure 1) able to bind and stabilize r(G_4_C_2_)_n_ G4‐forming sequences,^[22]^ leading to a significant reduction of both RNA foci and DPR protein formation. These results suggest that selectively targeting *C9orf72* r(G_4_C_2_)_n_ sequences could represent a novel potential treatment strategy for neurological disorders such as ALS/FTD. However, the structural details of the interaction of these compounds with their macromolecular target are unclear to date.

In this regard, computational methods proved to be extremely useful in filling the gaps when structural data are missing. For example, molecular dynamics (MD) simulations represent powerful computational tools that provide structural and dynamic insights at an atomistic level. Recent studies have employed this computational technique to examine binding interactions between G4 and potential therapeutic agents[^23^, ^24^] and evaluate ligand‐binding specificity using free binding energy calculations for G4 DNA.^[25]^

In this work, by applying *in silico* techniques, we have unraveled at the atomistic level the binding mode of the three aforementioned organic compounds. Moreover, to broaden the scope of our approach, we have investigated the binding to the selected G_4_C_2_ DNA and RNA sequences of an organometallic gold(I) compound, [Au(9‐methylcaffein‐8‐ylidene)_2_]^+^ (Au(TMX)_2_, Figure 1), previously reported to be a potent and selective stabilizer of distinct G4 structures,^[21]^ by coupling experimental techniques, such as FRET DNA melting assays, along with distinct computational methods, such as MD and metaD simulations.

## Results and Discussion

### Unraveling the Molecular Basis for the Activity of DB1246, DB1247, and DB1273

**Figure 2:**
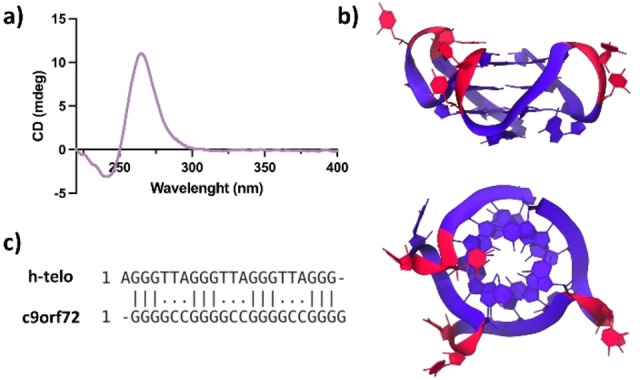
a) CD spectrum of the RNA‐G4 recorded in 20 mM potassium cacodylate buffer (pH 7.4). b) Side and top view of a representative structure of the RNA model obtained from MD simulations using cluster analysis. c) Sequence alignment between the selected human telomeric (h‐telo) G4 and our C9orf72 target sequence. The main differences reside in the composition of the G4 loops.

Structural data on RNA secondary structures are generally difficult to obtain. Since no structural data are available for RNA G4 formed by (G_4_C_2_)_n_ repetition, we have preliminarily recorded the CD spectra of r(G_4_C_2_)_3_G_4_, and the results obtained suggest the occurrence of a parallel folding of the strands (Figure 2A). This is in line with similar results obtained for repetitions of the *C9orf72* gene,^[10]^ and also reported for many RNA G4 structures.^[11]^ Thus, to obtain an atomistic model of r(G_4_C_2_)_3_G_4_ secondary structure, a homology modeling protocol has been employed (See Methods section).^[26]^ In particular, we have chosen as a template the parallel human telomeric structure with PDB ID 1KF1,^[27]^ since it is the one that more closely matches to our target sequence (Figure 2b). The main difference between the two sequences, as highlighted by the alignment shown in Figure 2C, are the following: i) in the 5’ side of the *C9orf72* sequence there is no A, but it starts with the second G; ii) the 3’ end of the target sequence has an additional G; iii) the TTA loops of the telomeric sequence are substituted by GCC loops. After building the model, we relaxed it under physiological conditions by performing a 1 μs‐long MD simulation. The system reached a stable conformation after about 400 ns, with most relevant fluctuations limited to the external GCC loop (Figure S1).

A series of three structurally related compounds (DB1246, DB1247, and DB1273, Figure 1), which have been shown to selectively bind r(G_4_C_2_)_n_, was identified using a DNA/RNA experimental screening approach.^[22]^ To investigate the mechanism of action of these compounds, in the absence of structural data, we have employed distinct *in silico* approaches. First, docking calculations were performed on the two available DNA conformations (AQP and NAN) and the most representative RNA structure obtained from the MD simulations. The best binding poses obtained for all investigated compounds are reported in Figure S2. Generally, in the DNA models, all molecules interact with the grooves of the G4s. The amidine moieties of all the compounds are predicted to be protonated at physiological pH, and this leads to establishing persistent H‐bonds with the negatively charged phosphate groups of the G4s. The preferred interaction with the grooves is also dictated by the fact that, in these conformations, the terminal G‐quartets are hindered by the loop cytosines. In the RNA model, terminal G‐quartets are not hindered by the flanking nucleobases (Figure 2B), and indeed, all investigated compounds display top‐stacking interactions with these quartets, as commonly performed by G4‐binders with parallel‐stranded G4s. Interestingly, the docking scores are in agreement with the experimental trend (observed preference for RNA over DNA) only for DB1246, while for DB1247 and DB1273, we observe a slightly better score for one or both DNA conformations (Table 1).

**Table 1:** Binding free energies (ΔG_b_, kcal/mol) as obtained from our docking calculations and those computed using the MM‐PBSA methods.^[28]^

Compound		Docking Score	ΔG_b_ MM‐PBSA
**DB1246**	**AQP**	−7.28	−20.33±2.47
	**NAN**	−7.79	−19.58±2.78
	**RNA**	−8.63	−25.83±2.89
**DB1247**	**AQP**	−9.20	−20.72±1.77
	**NAN**	−9.20	−30.97±3.31
	**RNA**	−8.13	−33.58±3.89
**DB1273**	**AQP**	−8.13	−23.81±2.39
	**NAN**	−9.05	−29.06±2.78
	**RNA**	−8.00	−30.67±2.81

**Figure 3:**
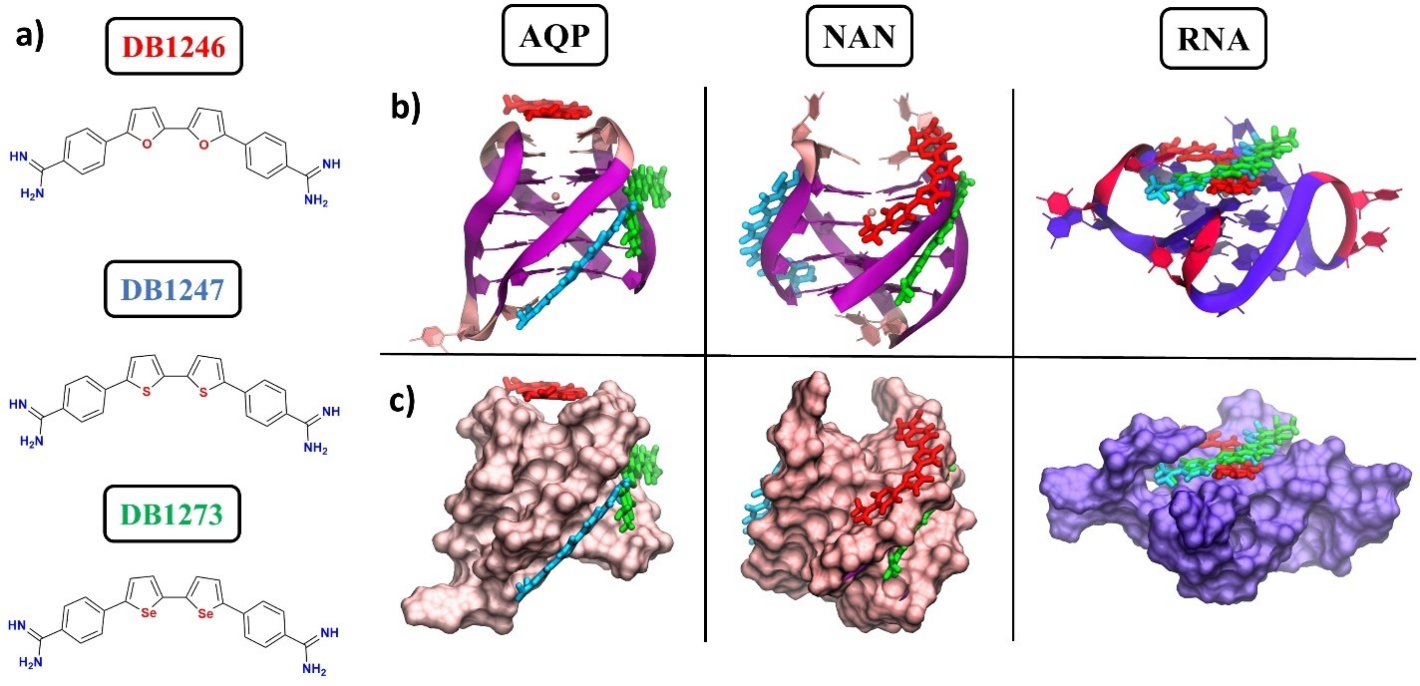
a) Structures of DB1246, DB1247, and DB1273. Representative structures obtained from the MD simulations performed on AQP, NAN, and RNA structures are shown as b) ribbons or c) surfaces. The investigated compounds DB1246 (red), DB1247 (cyan), and DB1273 (green) are shown with a sticks representation. In the DNA models (AQP and NAN), guanines and cytosines are shown in mauve and pink, respectively, while in the RNA model is highlighted in purple and red.

Next, considering the flexible nature of nucleic acids (especially RNA) and the current limitations shown by rigid docking calculations performed on these structures, the binding poses obtained from the previous docking calculations were validated by performing 500 ns‐long MD simulations. Representative structures obtained using cluster analysis are shown in Figure 3.

Interestingly, all binding poses are stable, even after relatively long MD runs of 500 ns, and all investigated compounds do not dissociate from the G4s in this time frame, thereby validating the docking protocol employed. In particular, in AQP, DB1246 tries to stack on top of the G‐quartet while the other two compounds lie in the same groove; in NAN, all three compounds stably sit inside different G4 grooves. We remind that terminal G‐quartets here are not accessible to accommodate the compounds to perform π–π stacking interactions. Finally, in RNA, all investigated binders perform top‐stacking interactions with the G‐quartets, with DB1247 and DB1273 showing similar binding poses. Generally, the binding is essentially driven by electrostatic interactions between the positively charged amidine motifs of the compounds and the negatively charged phosphate groups of the G4s (Figure S3).

These are generally higher in the case of the RNA G4, except for compound DB1273, where in the case of NAN, it stably occupies a narrow groove of the G4.

Next, to obtain a more reliable estimation of the affinity of the investigated compounds towards the nucleic acid structures, we have calculated the binding free energy (ΔG_b_) using the Molecular Mechanics Poisson Boltzmann Surface Area (MM‐PBSA) method (Table 1).^[29]^ This type of analysis, performed on equally spaced frames extracted from a stable part of the MD simulation trajectories, provides important insights into the noncovalent interactions (hydrophobic or electrostatic) between a small molecule and its receptor. Indeed, in contrast with what we have observed with the docking calculations, after refining the binding poses with the MD simulations, the MM‐PBSA method estimates for DB1247 and DB1273 a slightly higher ΔG_b_, for the r(G_4_C_2_)_3_G_4_ structure, now in better agreement with the experimental trend. Moreover, we also obtained a higher affinity for DB1247 than the other structurally related compounds, as also observed experimentally.^[22]^

**Figure 4:**
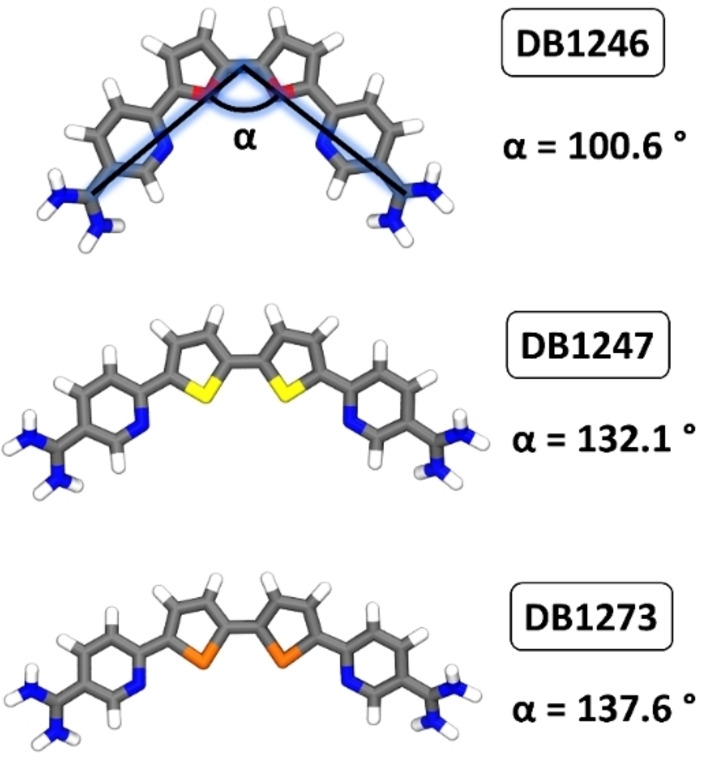
Optimized geometries, obtained by DFT calculations, of DB1246, DB1247, and DB1273. The angle between the two amidine carbons and the center of mass of the compounds is also shown.

Overall, our MD simulations have highlighted that similar binding poses between DB1247 and DB1273 are observed, especially in the case of AQP and RNA models (Figure 3b, c). Here, the only difference between these structurally related compounds relies on the nature of the heteroatoms present in the five‐membered ring. Oxygen, sulfur, and selenium are elements belonging to the 16^th^ group of the periodic table. Going down in the group, the atomic radius of the corresponding atom increases (1,71 Å for O, 2,14 Å for S, and 2,24 Å for Se).^[30]^ Indeed, the optimized structures of the three compounds show remarkable differences in the bending of these molecules (Figure 4), with DB1247 and DB1273 showing similar angles, which explains why these two molecules possess similar binding modes with the G4s.

### Investigating the Binding of Au(TMX)_2_

To further probe our investigational model, we expanded our methodology to include metal‐based entities. Several metal complexes have demonstrated promising G‐quadruplex binding properties, with some exhibiting greater efficacy in stabilizing non‐canonical DNA structures compared to purely organic molecules.[^15^, ^31^, ^32^] Notably, Au(I) N‐heterocyclic carbene (NHC) complexes, particularly those derived from benzimidazole‐like ligands, have recently attracted considerable attention due to their biological properties.^[33]^ These complexes can attain the stabilization of the Au(I) oxidation state, which is essential for their biological activity.[^34^, ^35^, ^36^, ^37^] Consequently, several Au(I) NHC complexes have been investigated for their interaction with human targets, including DNA‐G4 structures. Specifically, in 2014, the organogold compound Au(TMX)_2_ (Figure 1) derived from a xanthine‐like ligand^[38]^ exhibited potent and selective interactions with human G4s. While various modifications to the compound's scaffold have been reported in different studies,[^39^, ^40^, ^41^] none of the newly synthesized derivatives have surpassed the stabilization properties of Au(TMX)_2_. Furthermore, its G4 DNA targeting in human ovarian cancer cells was indirectly confirmed using mass spectrometry and pharmacological approaches.^[42]^ Overall, Au(TMX)_2_ serves as an ideal benchmark for G4‐targeting, prompting our decision to incorporate it into our investigations to elucidate the molecular recognition process of ALS‐derived G4 sequences, whether from DNA or RNA.

Thus, we have performed a series of FRET DNA melting experiments using both the RNA and DNA sequences under investigation. Owing to the high guanine content, elevated melting temperatures (T_m_) were observed when recording the G4‐meltings in a 60 mM potassium cacodylate buffer (pH=7.4), a commonly employed medium for such experiments. However, additional stabilization of these curves by the gold(I) NHC complex would have led to their shifts beyond 100 °C, resulting in incomplete melting profiles. Consequently, before investigating the compound‐stabilization properties, a preliminary adjustment of the buffer‘s concentration was necessary. Specifically, 10 mM and 20 mM potassium cacodylate buffers (pH=7.4) were evaluated alongside the standard 60 mM solution.

**Figure 5:**
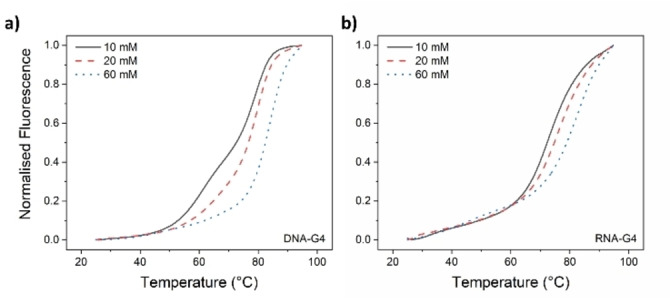
DNA‐ (a) and RNA‐G4 (b) melting profiles were recorded in 10 (solid black lines), 20 (dashed red lines), and 60 mM (dashed blue lines) potassium cacodylate buffer.

**Figure 6:**
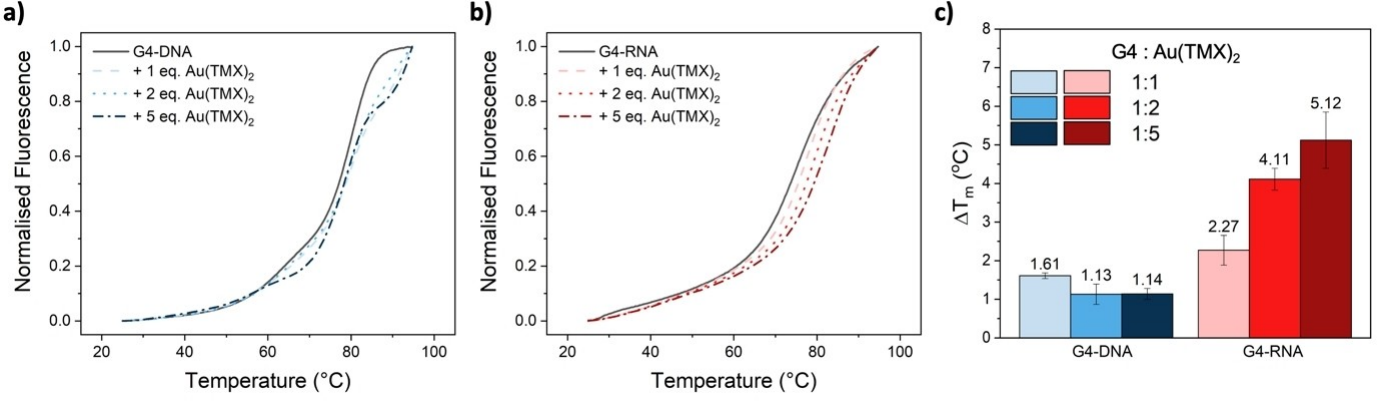
a, b) Melting profiles recorded in 20 mM potassium cacodylate buffer (pH=7.4) of G4‐DNA and ‐RNA alone (black solid lines) and in the presence of 1, 2, and 5 eq. of Au(TMX)_2_; c) increases in the melting temperatures (ΔT_m_) of the selected G4s in the presence of increasing amounts of Au(TMX)_2_.

As depicted in Figure 5, a significant shift towards lower temperatures is evident when reducing the concentration from 60 mM (dashed blue lines) to 10 mM (solid black lines) potassium cacodylate buffer (pH=7.4), consistent with the anticipated lower stabilization of the polynucleotide.^[43]^ However, while the melting profile shape of the RNA‐G4 appears unaffected by the varying concentrations, the use of a 10 mM potassium cacodylate buffer (pH=7.4) induces the formation of a shoulder in the melting trace between 45 and 65 °C (black line in Figure 5a), which is not observed with the other two buffers. This suggests an incomplete G4‐folding under these conditions, as also supported by ^1^H NMR studies conducted by the Plavec group.^[8]^ Consequently, a 20 mM potassium cacodylate buffer (pH=7.4) was selected for our studies, yielding reasonable T_m_ values of approximately 77 °C and 74 °C for DNA and RNA‐G4, respectively. The presence of the Au(I) NHC complex weakly stabilizes the DNA‐G4 sequences, raising their T_m_ by approximately 1.6 °C in a 1 : 1 ratio. This effect remains consistent with increasing amounts of the Au(I) compound up to a 1 : 5 stoichiometry, as illustrated in Figure 6a, c.

While being a potent G4 stabilizer for parallel and hybrid‐mixed (3 : 1) folded DNA‐G4,^[41]^ the antiparallel folding of the DNA sequence under investigation appears to deter the interaction of Au(TMX)_2_ with the DNA‐G4. Conversely, exposure of the RNA‐G4 to the Au(I) NHC complex leads to a notable increase in the G4 melting temperature, with the latter rising from 1 to 5 equivalents of Au(TMX)_2_ up to approximately 5.0 °C (Figure 6b, c). Nevertheless, these increases are significantly less pronounced than those reported for Au(TMX)_2_ in the presence of other G4 structures, as well as those observed by Simone et al. for the *C9orf72* G4s with the previously mentioned organic ligands, DB1246, DB1247, and DB1273.^[22]^

**Figure 7:**
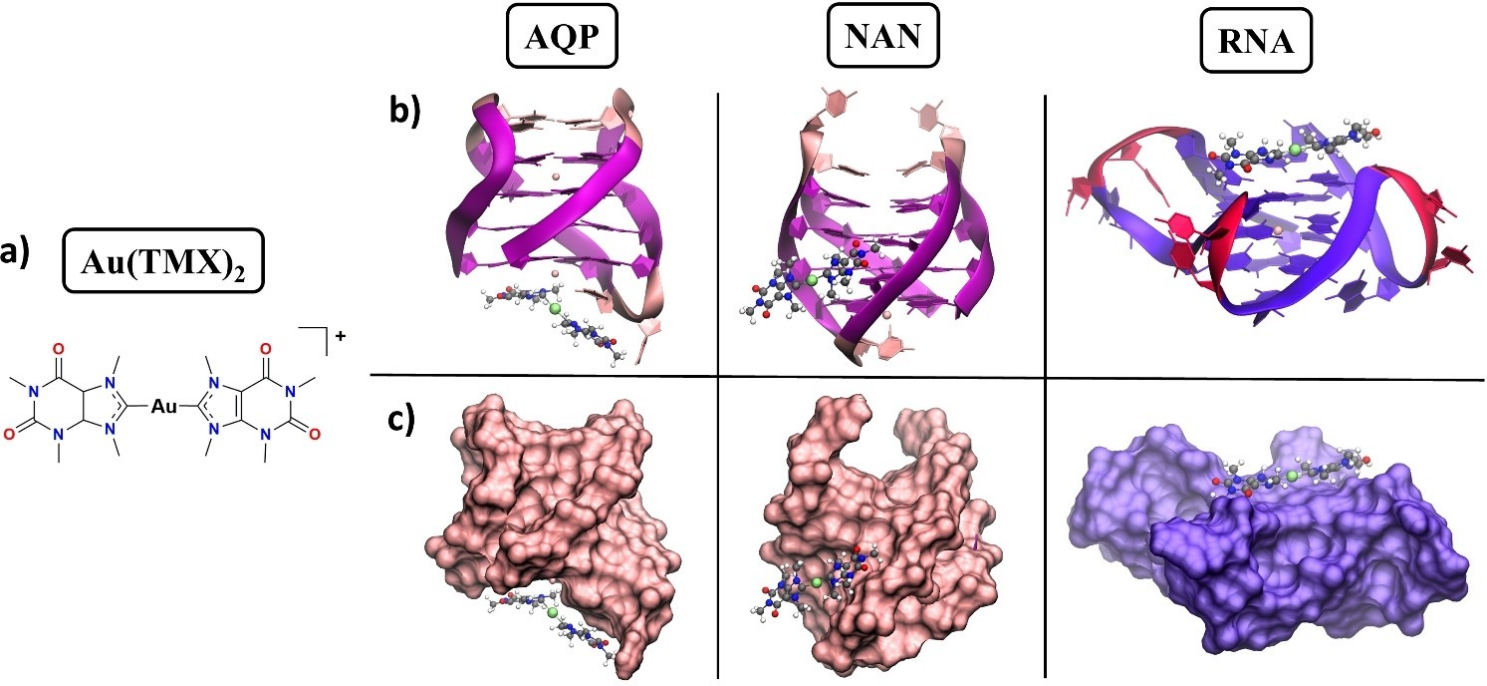
a) Molecular structures of the Au(TMX)_2_ complex. Binding poses extracted from the MD simulations performed on AQP, NAN, and RNA structures are shown as b) ribbons or c) surfaces. Au(TMX)_2_ is shown with a stick representation, while in the DNA models (AQP and NAN), guanines and cytosines are shown in mauve and pink, and in the RNA model are highlighted in purple and red, respectively.

Hence, to rationalize these discrepancies, we decided to shed light on the G4‐binding mode of Au(TMX)_2_ by applying a series of computational techniques. First, we performed docking calculations with our three *C9orf72* models (AQP, NAN, and RNA) using the same protocol applied previously (Figure S4). Next, the obtained docking poses were validated by performing 500 ns‐long MD simulations, and representative structures are shown in Figure 7.

These have highlighted that Au(TMX)_2_ performs stacking interactions with the exposed G‐quartets of the RNA model, while the binding poses identified in both the DNA models were revealed not to be stable, validating the negligible melting temperature increases observed with the FRET melting assays. In particular, in the binding with AQP, Au(TMX)_2_ performs top‐stacking interactions with a terminal G‐quartet, while with NAN, it establishes a groove binding mode, very stable along the MD simulation. This confirms the well‐known general lack of accuracy shown by docking calculations, in particular when involving metal compounds, in finding their binding poses to biological macromolecules, along with the necessity to refine these poses using other computational techniques.^[44]^ Finally, these qualitative observations are consistent with the ΔG_b_ values obtained with the MM‐PBSA method (Table 2), where we noticed a higher binding affinity toward the RNA sequence.

**Table 2:** Binding free energies (ΔG_b_, kcal/mol) as obtained from our docking calculations and those computed using the MM‐PBSA methods.^[28]^

Compound		Docking Score	ΔG_b_ MM‐PBSA
**Au(TMX)_2_**	**AQP**	−5.57	−13.4±2.54
	**NAN**	−5.45	−11.96±2.80
	**RNA**	−4.77	−18.57±2.22

**Figure 8:**
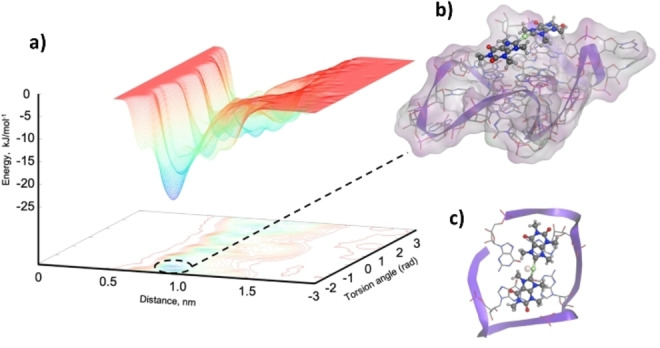
a) Representative metaD free energy surface (FES) based on a two CV calculation. CVs correspond to the distance (nm) of Au(TMX)_2_ and K^+^ at the center of the G4 tetrad and torsional rotation (rad). The lowest energy state is highlighted with b), showing the position of Au(TMX)_2_ in regard to this energy minima. c) a top view of the position of Au(TMX)_2_ on the upper tetrad, showing how the ligand maximizes the stacking interactions with guanine bases (G4 is shown in the ribbon and stick representation, with a molecular surface colored according to lipophilicity (green: lipophilic, purple: hydrophilic). Au(TMX)_2_ is shown in ball and stick representation.

In light of these encouraging results, we have decided to further refine the binding pose obtained by our classical MD simulations of Au(TMX)_2_ with our RNA model using metaD. Such a technique has been proven successful in predicting the ΔG_b_ value of binding for organic and metal complexes binding nucleic acid structures.[^21^, ^45^] In particular, to accurately estimate the Gibbs free energy of binding, 20 runs were performed where Au(TMX)_2_ was allowed to find the most favorable binding pose around the entire G4 model. Thus, two collective variables (CV) were taken into account: the first CV (CV1) considered the distance between Au^+^ and the potassium ion closest to the binding tetrad; the second CV (CV2) took into account rotations of the torsion angle between Au(TMX)_2_ and the uppermost tetrad (see Methods Sections). In the vast majority of the metaD runs, Au(TMX)_2_ binds to the freely accessible top quartet, performing several but energetically comparable stacking interactions (Figure S5), as observed in the aforementioned MD simulations and a previous study.^[21]^ Interestingly, metaD simulations allowed an extensive sampling of the free energy surface, overcoming in this way the limitations of classical MD simulations. This translates into binding free energy (ΔG_metaD_) of −5.52±0.76 (Figure 8), which is in agreement with the experimental energy of binding (ΔG_exp_) of −6.51±0.43, as obtained by applying a literature‐established protocol.^[21]^ Interestingly, as for the melting temperature increase, both the binding free energies are lower than previously observed for the interaction of Au(TMX)_2_ with other G4 structures, such as h‐telo and *cKIT1*.^[21]^ This is probably due to the lack of a defined binding pocket in our RNA model, leading to a plethora of very similar binding poses performing top stacking interactions with the whole top G‐tetrad (Figure S5).

## Conclusions

Developing potent and selective G4 binders has always been challenging due to the high degree of polymorphism and similarity of G4 structures. However, some differences arise between DNA and RNA G4s regarding conformations. Thus, it is possible to design small molecules that can selectively target one of the latter. Considering HRE′s key role in familial ALS and FTD pathogenesis, research efforts nowadays focus on developing small molecules that can selectively bind the RNA G4 structures and, thus, hamper RNA foci and DPR formation.

In this context, three structurally related compounds (DB1246, DB1247, DB1273, Figure 1) able to target r(G_4_C_2_)_n_ were previously identified using a DNA/RNA parallel screening method.^[22]^ Here, by applying distinct computational techniques, we are shedding light on the binding mode of these molecules along with an Au^I^/N‐heterocyclic carbene complex. Our results furnished precious insights into their mechanisms of action, highlighting that the binding of all the investigated compounds is mainly driven by electrostatic interactions between the positively charged amidine moieties (or the charged Au^+^ ion) and the negatively charged phosphates groups. Furthermore, RNA recognition is guided by top‐stacking interactions, which is a typical interaction mode displayed by small molecules with parallel G4s. Similar binding modes were predicted using *in silico* techniques for TMPyP4, a well‐known potent G4 binder,^[46]^ towards r(G_4_C_2_)_n_,^[47]^ further confirming our hypothesis.

**Figure 9:**
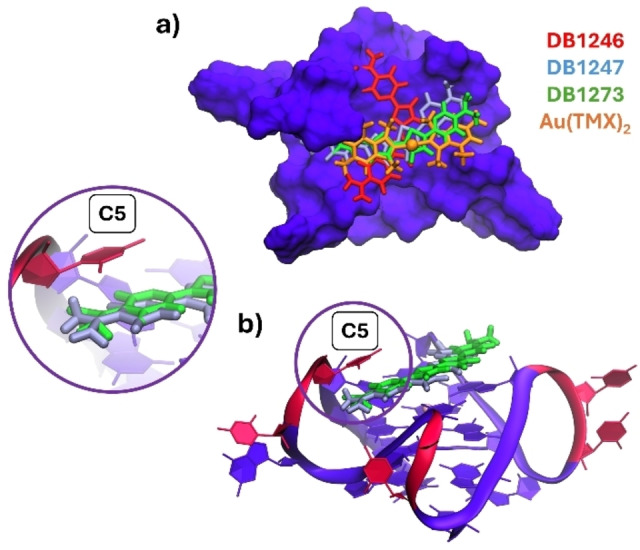
a) Overlap of representative structures extracted from the MD simulations using cluster analysis. DB1246, DB1247, DB1273, and Au(TMX)_2_ are shown in red, blue, green, and orange stick representations. b) Side view of the additional stacking interactions provided by C5 with the compound DB1247 and DB1273. Guanine and cytosine residues are shown in purple and red, respectively.

Interestingly, we have noticed that the antiparallel folding of the investigated DNA sequences (AQP and NAN) hampers the interaction of Au(TMX)_2_. On the other hand, the parallel fold of the RNA model leads to a stronger interaction involving top‐stacking interactions with the accessible G‐tetrads, showing an increase in the G4 melting temperature up to approximately 5.0 °C. However, due to the lack of a defined binding pocket, the interaction is not specific, leading to a low binding energy value of −6.51±0.43, which remarkably agrees with the one obtained by the metaD simulations of −5.52±0.76. Such value is, however, lower than previously observed for the interaction of Au(TMX)_2_ with other G4 structures. As Au(TMX)_2_ extensively samples the external G‐quartet of the RNA model, it leads to several, but energetically similar, binding poses (Figure S5). It should be noted that Au(TMX)_2_ does not possess side arms that can extend to the G4 grooves like the porphyrin and strong stabilizer TMPyP4, anchoring the compound to the G4 tetrad and tightening the binding through electrostatic interactions with the negatively charged grooves.^[21]^ Lastly, by comparing the binding poses of the four molecules investigated in this study, we can hypothesize that the bent structure of DB1246, DB1247, and DB1273 leads to better recognition of the top tetrad, as compared to the linear geometry possessed by Au(TMX)_2_ as can be seen by overlapping of all the best representative structures (extracted from MD simulations, Figure 9a). Moreover, this geometry also leads in the case of DB1247 and DB1273 (which possess a very similar binding angle, Figure 4) to a further stabilization with additional stacking interactions involving C5, a cytosine from one nearby loop (Figure 9b). It could be speculated that this additional interaction contributes to the most favorable binding energy observed for these compounds toward the RNA model (Table 1).

Thus, we have dissected, for the first time, with an atomistic level of detail, the molecular basis for the selectivity of distinct *C9orf72* G4‐binders. Therefore, our results could be exploited to guide the rational design of novel selective compounds that display selectivity towards RNA (G_4_C_2_)_n_ repetitions, paving the way toward developing potent and selective G4‐binders for treating neurodegenerative diseases.

## Methodology

### Model Building

We have built three G4 models. The two d(G_4_C_2_)_3_G_4_ were obtained from the Protein Data Bank (pdb ID 2N2D and 5OPH),[^8^, ^9^] while d(G_4_C_2_)_3_G_4_ was obtained following a homology modeling procedure (see next section). Following the nomenclature of the original papers, these two structures were solved using solution‐state NMR and were named in the text as AQP (acidic and quenching) and NAN (neutral and annealing). Among these, NAN is thermodynamically favored, while AQP is kinetically favored under slightly acidic conditions. The structures of DB1246, DB1247, and DB1273 were optimized by DFT calculations using the B3LYP functional and the 6‐31G* basis set with the Gaussian16 program.^[48]^ In the case of Au(TMX)_2,_ the Stuttgart/Cologne Group SDD basis sets pseudopotential was used.^[49]^

### Homology Modelling for RNA (G_4_C_2_)_3_G_4_

Since no structural data are available to date for RNA G4s composed of GGGGCC repetitions, a homology modeling protocol was employed to obtain a starting model of its secondary structure.^[26]^ Considering that the circular dichroism spectra of the investigated sequence, r(G_4_C_2_)_3_G_4_, exhibit the typical trait of a parallel arrangement (Figure 2A), in accordance with the literature,^[10]^ we restricted our search to sequence folding with parallel orientation. Thus, we have chosen as a template for the homology modeling a typical parallel human telomeric structure (PDB ID 1KF1)^[27]^ (Figure 2a). The DNA structure was converted into an RNA sequence using the “Mutagenesis Wizard” tool of the PyMOL software. Moreover, in order to obtain a relaxed RNA structure, we have performed a 1 μs‐long MD simulation.

### Docking Calculations

Docking calculations were performed using AutoDock4.2.^[50]^ All files were converted into the AutoDock format using AutoDockTools.^[51]^ For all investigated systems we have selected a docking box large enough to include the whole G4 structure. Geister‐Marsili partial charges were attributed to the investigated compounds and nucleic acid structures. As typically performed in docking calculations, the receptor structure was kept rigid while the binders were flexible, i. e. a conformational search was allowed around putative single bonds. For each docking, 50 runs were performed, using the Lamarckian Genetic algorithm and a maximum of 2.5 ⋅ 10^6^ energy evaluations. The most representative binding pose was finally extracted, i. e. the nucleic acid‐binder complex structure showing the lowest binding free energy of the most populated cluster.

### Molecular Dynamics (MD) Simulations

MD simulations were performed with the GROMACS 2021.2 software package.^[52]^ The most tested force field for nucleic acids, namely the Amber ff99+bsc0+OL15 FF^[53]^ and Amber ff99+bsc0+χOL3 FF,^[54]^ were used for DNA and RNA sequences, respectively. All systems were embedded in a 15 Å layer of TIP3P water molecules leading to a box of 64 Å×64 Å×64 Å, counting up to 25k atoms. The charge of the system was neutralized, and additional K^+^ Cl^−^ ions were added to achieve a physiological salt concentration of 0.150 M. All topologies were built with Ambertools 23^[55]^ and were subsequently converted in a GROMACS format using the software parmed. The partial charges of all the studied binders were calculated by performing geometry optimizations at the Hartree‐Fock (B3LYP in the case of Au(TMX)_2_) level of theory, using the 6‐31G* basis set (SDD in the case of Au(TMX)_2_), with the Gaussian16 program package.

In all MD simulations, we have used a soft equilibration protocol, as described previously.[^56^, ^57^] First, the systems went initially through an energy minimization employing a steepest descent algorithm considering a force convergence criterion of 1000 kJ/mol ⋅ nm^2^. Next, the systems were annealed from 0 to 300 K using a temperature gradient of 50 K every 2 ns, with a total of 12 ns. Here, the G4s and the binders were subjected to harmonic position restraints with a force constant of 1000 kJ/mol nm^2^, while water molecules and Na^+^ ions were allowed to move. Once the target temperature of 300 K was reached, 20 ns of NPT simulations were conducted to stabilize the pressure to 1 bar by using a Berendsen barostat. The same restraints used in the heating phase were retained in this phase. Temperature control at 300 K was achieved by stochastic velocity rescaling thermostat.^[58]^ Afterward, the barostat was switched to Parrinello‐Rahman,^[59]^ and the restraints on the atoms of the G4s were restricted only to the backbone atoms. These restraints were then gradually decreased into three consecutive runs of 20 ns each, during which the force constant was set to 1000, 250, and 50 kJ/mol nm^2^, respectively. Thus, after a long equilibration protocol of ∼100 ns, all the position restraints were released, and all production runs were performed for 500 ns for each of the models. Finally, productive MD simulations were performed using the isothermal‐isobaric ensemble (NPT). The particle mesh Ewald method was used to account for long‐range electrostatic interactions with a cutoff of 10 Å, and a LINCS algorithm was used to constrain the bonds involving hydrogen atoms. An integration time step of 2 fs was used in all simulations.

### Molecular Mechanics Poisson–Boltzmann Surface Area (MM‐PBSA)

The Amber 18 tool MM_PBSA.py^[60]^ was used to perform Molecular Mechanics Poisson–Boltzmann Surface Area (MM‐PBSA) free energy calculations, taking 100 frames from the last 100 ns of the MD trajectories. The conformational entropic component of the free energy was not considered since it was previously suggested that this term does not improve the quality of the results using the MM‐PBSA method.^[61]^ The VMD program was used to visualize structures and MD trajectories.^[62]^

### Metadynamics (metaD) Simulations

Free energy calculations were performed on the G4‐RNA model structure with [Au(9‐methylcaffein‐8‐ylidene)_2_]^+^, Au(TMX)_2_. The Au(TMX)_2_ cation was placed approximately 10 Å above the upper surface of the G4‐RNA structure. All simulations were run using the GROMACS 2021.2 – Plumed 2.9.0 software, using a recently published procedure.^[21]^ Twenty well‐tempered metaD simulations were run for 1.5 ⋅ 10^8^ steps with a 1 fs time step (for a total of 150 ns), for a total of 3μs simulation time. MetaD was implemented using the Plumed plugin^[63]^ for GROMACS with two collective variables (CVs). CV1: a distance was set up between the Au^+^ of the metal complex and the K^+^ of the top tetrad of the G4 model. CV2: a torsion angle, defined by the two carbons on either side of the gold center for Au(TMX)_2_ (C2 and C4) and the N1 of G2 and N1 of G14 of the G4‐RNA. The computational parameters were set with a Gaussian height of 0.05 kJ/mol, a Gaussian width of 0.025 Å, and a torsion angle of 0.08 rad. Gaussian functions were added every 2000 steps (2 ps), giving a deposition rate of 0.025 kJ/(mol.ps). The bias factor was set to 12; thus, the DT was 3600 K. Free energy surfaces (FES) were obtained for each run with the ΔG taken from the lowest energy minima of each FES. Statistical outliers were removed, and the average ΔG was calculated (n=16).

### Analysis

MD trajectories were analyzed and visualized using the VMD software.^[62]^ Root‐mean‐square deviation (RMSD) analysis was performed with the tools implemented in GROMACS 2021.2.^[52]^ Cluster analysis of all the MD trajectories was carried out using the g_cluster tool.^[52]^ All analyses were done on the equilibrated part of the trajectories (200–500 ns).

### Compound Synthesis

All the reagents and solvents were commercial and used without any further purification. Au(TMX)_2_ was prepared according to literature‐reported protocols,[^38^, ^39^] assessing its purity above 95 % via elemental analysis.

### Circular Dichroism (CD) Spectra

CD spectra were recorded on a Jasco J‐715 spectropolarimeter at 25 °C. For this experiment, we used the G4‐folded 5′‐GGGGCCGGGGCCGGGGCCGGGG‐3′ oligodeoxynucleotide (ODN) sequence at 2 μM concentration. The parameters were the following: range 400–220 nm, response: 0.5 s, accumulation: 3, speed 200 nm/min. ODN was resuspended in Tris‐EDTA buffer (pH 7.4) to yield a 1 mM stock solution expressed in strand units. To obtain the 2 μM concentration, the ODN was diluted in potassium cacodylate buffer (20 mM, pH=7.4) and folded in its G4 topology heating at 90 °C for 5 min followed by slowly cooling to room temperature.

### FRET Thermal Stability Assays

FRET experiments were performed on a 96‐well plate using an Applied Biosystems® 7500 Real‐Time PCR cycler equipped with a FAM (6‐carboxyfluorescein) filter (λ_ex_=492 nm; λ_em_=516 nm). DNA and RNA sequences (FAM‐GGGGCCGGGGCCGGGGCCGGGG‐TAMRA) were purchased from IDT (Integrated DNA Technologies) in HPLC purity grade with FAM and TAMRA (6‐carboxy‐tetramethylrhodamine) as probes. Lyophilized DNA and RNA oligonucleotides (ONs) were resuspended and stored in a freezer in 1.0 mM Tris‐HCl and Tris‐EDTA (10 mM Tris‐HCl, 1.0 mM EDTA), respectively, both at pH 7.4. To afford the G4 folding, FRET ONs stock solutions were diluted to the desired concentration using 20 mM potassium cacodylate buffer (pH 7.4) and then heated to 90 °C for 5 min, followed by cooling to room temperature. In the final 30 μl solutions, ONs final concentration was set to 0.2 μM. To evaluate the stabilization and the possible concentration‐dependent effects of Au(TMX)_2_, three different [DNA]/[metal complex] ratios were used, 1 : 1, 1 : 2 and 1 : 5.

Au(TMX)_2_ was dissolved in DMSO to give 1 mM stock solution and then diluted with the buffer reaching a total percentage of DMSO never above 0.1 %. Experimental binding free energy (ΔG_exp_) values were obtained from folded fractions profiles obtained from the FRET melting curves, according to a literature‐established protocol.^[21]^

## Conflict of Interests

The authors declare no conflict of interest.

1

## Supporting information

As a service to our authors and readers, this journal provides supporting information supplied by the authors. Such materials are peer reviewed and may be re‐organized for online delivery, but are not copy‐edited or typeset. Technical support issues arising from supporting information (other than missing files) should be addressed to the authors.

Supporting Information

## Data Availability

The data that support the findings of this study are available from the corresponding author upon reasonable request.
