# Supplementary material for: Unraveling the Molecular Basis for G‐Quadruplex‐Binders to ALS/FTD‐Associated G4C2 Repeats of the C9orf72 Gene
Source: Chembiochem. 2025 Jan 20;26(8):e202400974. doi: 10.1002/cbic.202400974 (PMC12007073; doi:10.1002/cbic.202400974)
Supplement: Supplementary file 1 — Supporting Information [file CBIC-26-e202400974-s001.pdf]

ChemBioChem

Supporting Information

Unraveling the Molecular Basis for G-Quadruplex-Binders to ALS/FTD-Associated G4C2 Repeats of the *C9orf72* Gene

Luisa D'Anna, Darren Wragg, Daniela Mauro, Simona Rubino, Alessio Terenzi, Giampaolo Barone, Sophie R. Thomas, Angela Casini, Riccardo Bonsignore,* and Angelo Spinello*

SUPPORTING INFORMATION

Unraveling the molecular basis for G-quadruplex-binders to ALS/FTD-associated G4C2 repeats of the *C9orf72* gene

Luisa D'Anna,[†a] Darren Wragg,[†b] Daniela Mauro,[a] Simona Rubino,[a] Alessio Terenzi,[a] Giampaolo Barone,[a] Sophie Thomas,[c] Angela Casini,[b] Riccardo Bonsignore,*[a] Angelo Spinello*[a]

[a] Department of Biological, Chemical, and Pharmaceutical Sciences, and Technologies, Università di Palermo, Viale delle Scienze Edificio 17, 90128 Palermo, Italy

[b] Chair of Medicinal and Bioinorganic Chemistry, School of Natural Sciences, Department of Chemistry, Technical University of Munich (TUM), Lichtenbergstr. 4, 85748 Garching b. München, Germany

[c] Department of Inorganic Chemistry, University of Vienna, Währinger Straße. 42, Vienna, Austria

Figure S1 to S5 Pag. 2-6

Supplementary Figures

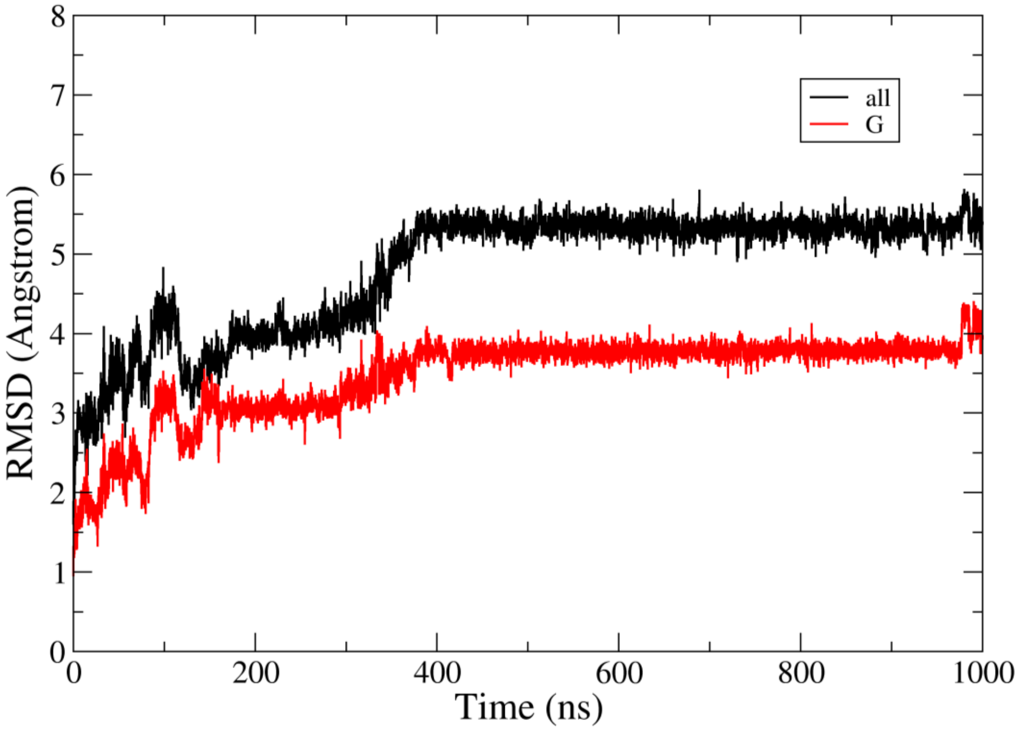

Figure S1. Root mean square deviation (RMSD, Å) *vs.* MD simulation time (ns) of the RNA model obtained via homology modeling, obtained considering all nucleotides (black line) and only the guanine residues (red line).

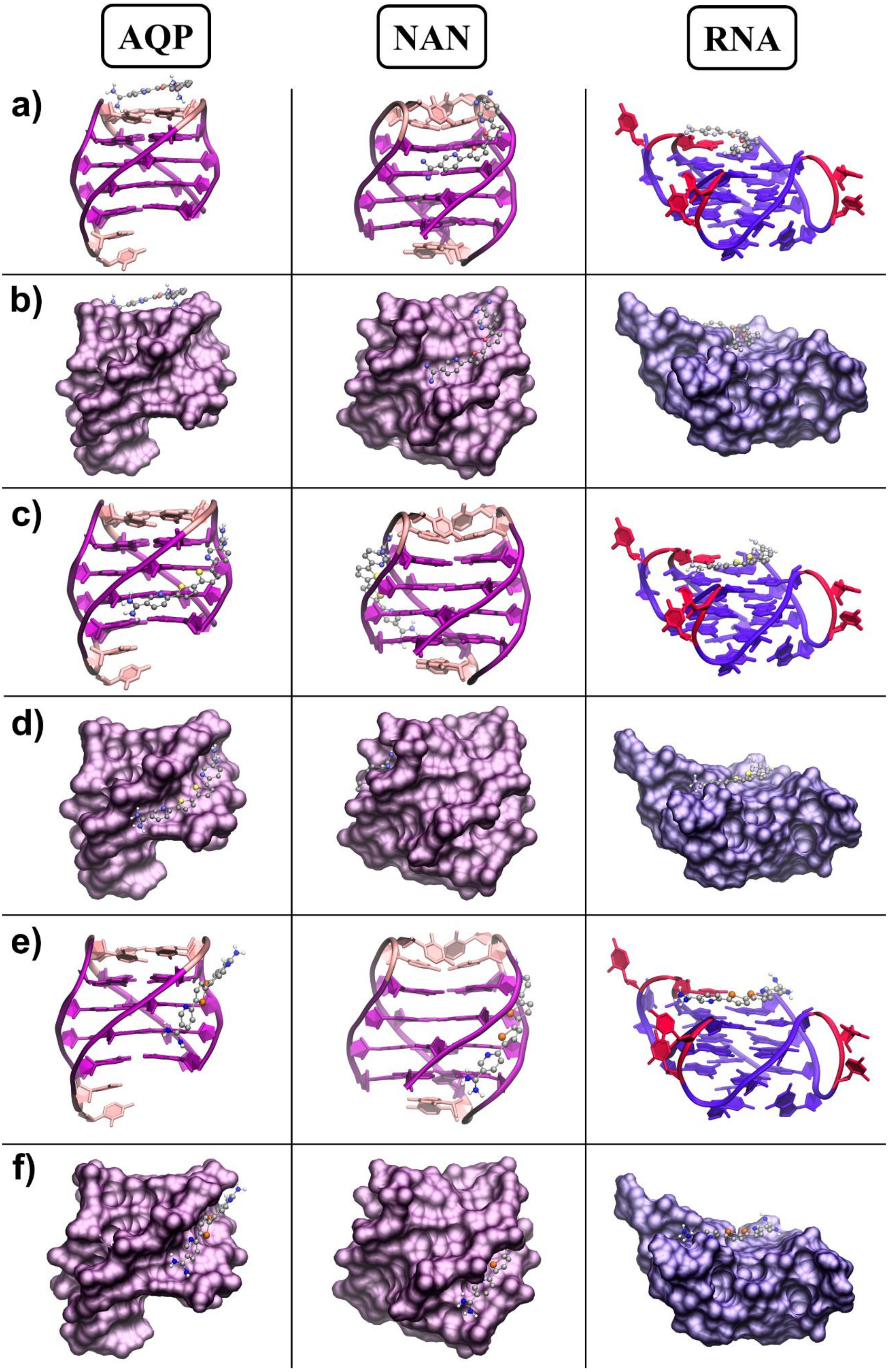

Figure S2. Binding poses obtained from the docking calculations performed on AQP, NAN, and RNA structures for the compounds DB1246 (a, b), DB1247 (c, d), and DB1273 (e, f). In the DNA models (AQP and NAN) guanines and cytosines are shown in mauve and pink, respectively, while in the RNA model are highlighted in purple and red, respectively.

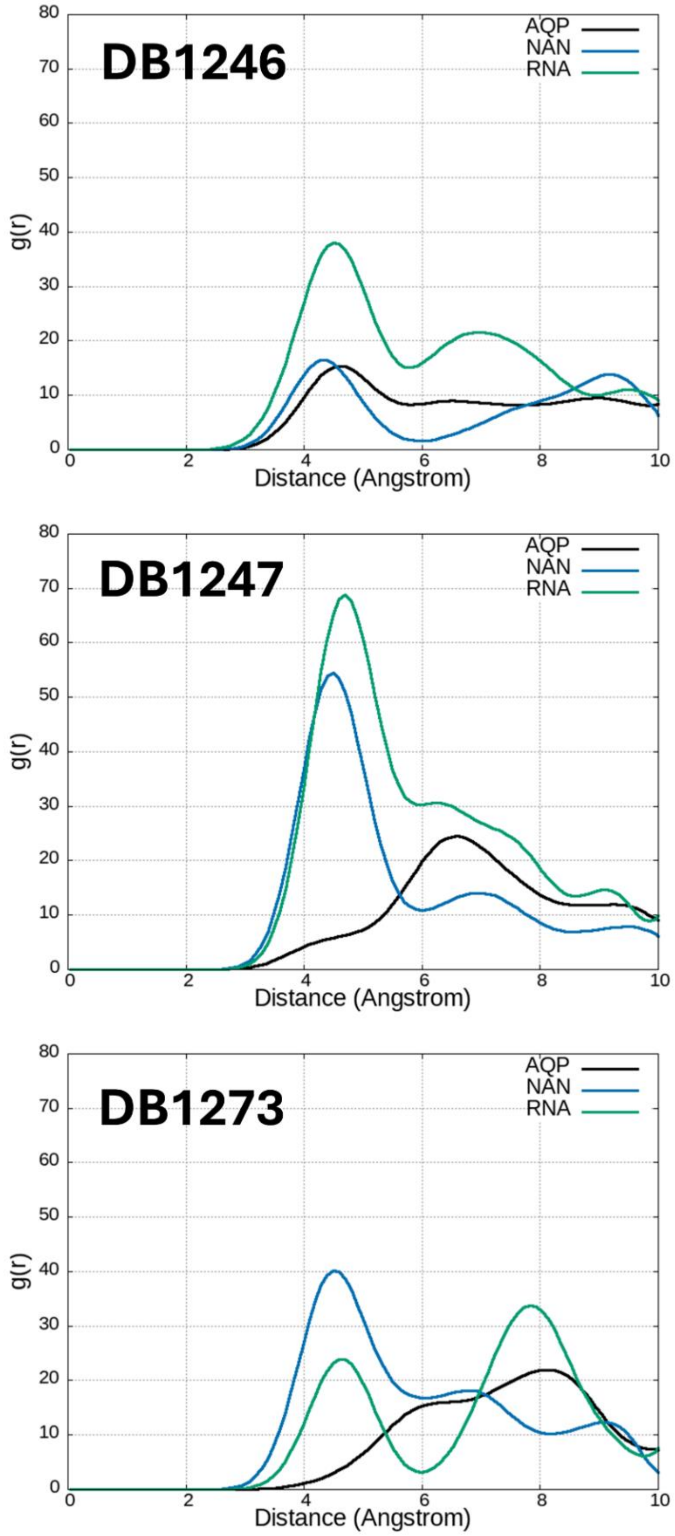

Figure S3. Radial Distribution Function, g(r), of the distance between the positively charged amidine moieties (centered on the nitrogen atom) of the investigated compounds (DB1246, DB1247, and DB1273) and the phosphorous atom of the negatively charged phosphate groups. Peaks centered at about 4 Å are indicative of H-bond formation.

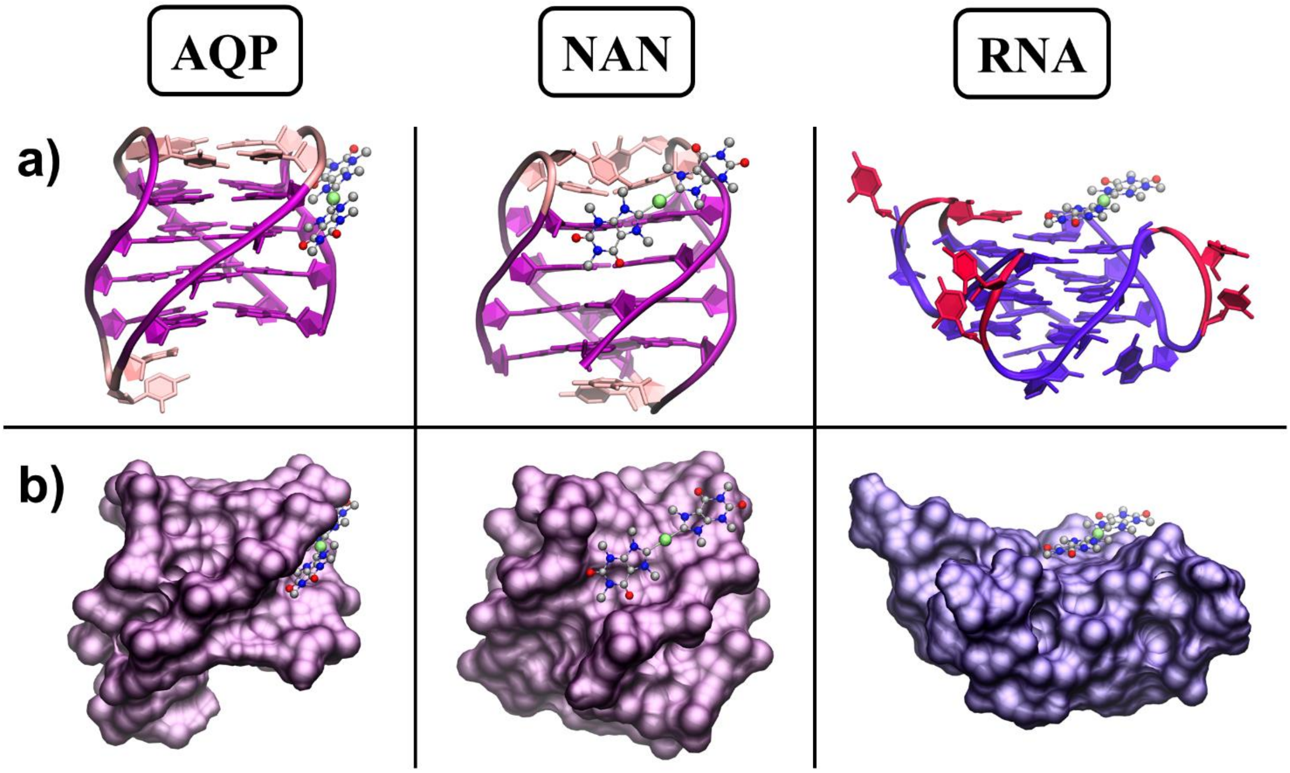

Figure S4. Binding poses of $Au(TMX)_2$ extracted from the docking calculations performed on AQP, NAN, and RNA. In a), the receptors are shown in ribbon representation, while in b), they are shown as surfaces. In the DNA models (AQP and NAN), guanines and cytosines are shown in mauve and pink, respectively, while in the RNA model, they are highlighted in purple and red.

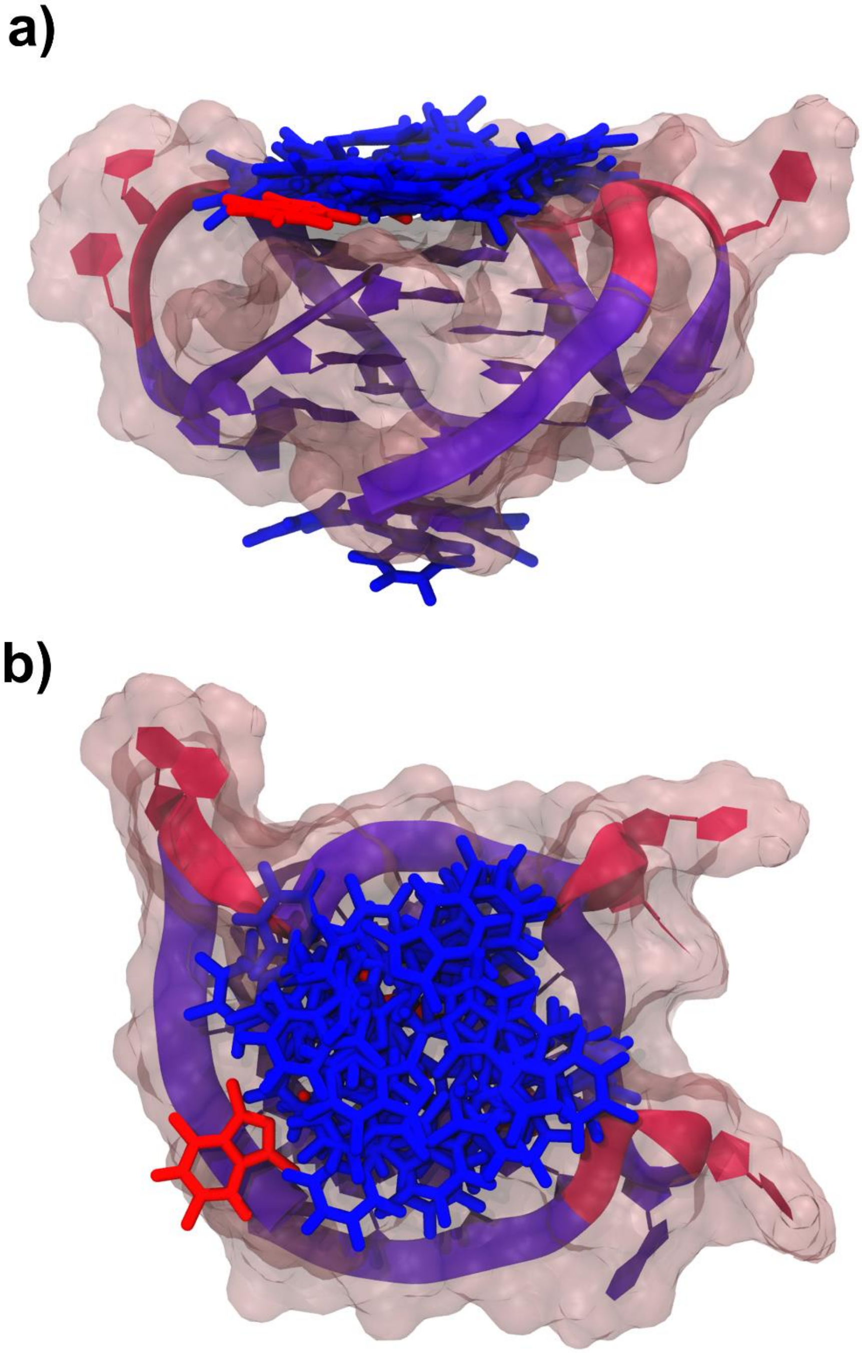

Figure S5. a) Side and b) top view of representative binding poses (blue) of the minimum energy structures, as extracted from 20 metadynamics (metaD) runs. The binding pose obtained from a cluster analysis of the classical MD trajectory is also shown in a red stick representation. $AuTMX_2$ is shown in licorice, while guanine and cytosine residues are shown in purple and red, respectively.
